# Mixed effects modelling of excess mortality and COVID-19 lockdowns in Thailand

**DOI:** 10.1038/s41598-024-58358-3

**Published:** 2024-04-08

**Authors:** Anna Christine De Padua Durante, Rutcher Lacaza, Pamela Lapitan, Nishtha Kochhar, Elaine S. Tan, Milan Thomas

**Affiliations:** 1 Economic Research and Development Impact Department, Asian Development Bank, Mandaluyong, Philippines; 2 Department of Economics, Georgetown University, Washington, USA

**Keywords:** Health policy, Public health

## Abstract

Accurate mortality data are critical for understanding the impact of COVID-19 and learning lessons from crisis responses. But published statistics risk misrepresenting deaths due to limited testing, underreporting, and lack of subnational data, especially in developing countries. Thailand experienced four COVID-19 waves between January 2020 and December 2021, and used a color-coded, province-level system for lockdowns. To account for deaths directly and indirectly caused by COVID-19, this paper uses mixed effects modelling to estimate counterfactual deaths for 2020–2021 and construct a monthly time series of provincial excess mortality. A fixed effects negative binomial and mixed effects Poisson model both substantiate other studies’ estimates of excess deaths using subnational data for the first time. Then, panel regression methods are used to characterize the correlations among restrictions, mobility, and excess mortality. The regressions show that mobility reductions modestly curbed mortality immediately upon imposition, suggesting that aversion of non-COVID deaths was a major aspect of the lockdowns’ effect in Thailand. However, the estimates are imprecise. An auto-regressive distributed lag model suggests that the effect of lockdowns was through reduced mobility, but the effectiveness appears to have varied over the course of the pandemic.

## Introduction

The World Health Organization defines a COVID-19 death as one in which COVID-19 is the underlying cause, but there is much international variation in how those deaths are defined and recorded, making it difficult to use official death counts for cross-national comparisons. As of January 2024, there were 7 million deaths according to official counts, but according to *The* *Economist’s* model estimates (accessed January 27, 2024) there were 28.5 million excess deaths worldwide due to COVID-19. The large disparity between official COVID-19 deaths and excess deaths exists for two main reasons. First, official counts typically define COVID-19 deaths narrowly, focusing on those directly caused by COVID-19 infection and omitting the indirect impacts on mortality through changes in access to emergency services, health-seeking behavior, government policy, and other complications. Second, testing, and administrative capacity constraints may prevent authorities from accurately estimating deaths, whether directly or indirectly caused by COVID-19^1^.

Excess deaths are a truer measure of the toll of the pandemic^2–4^ but the statistical models used to estimate them have limitations in estimating excess deaths due to COVID-19. They extrapolate excess mortality for countries with missing or unreliable data based on an algorithm trained on data from countries with reliable mortality data, which tend to be developed countries. Underlying modelling assumptions may be less appropriate for developing countries. This makes it critical to estimate excess mortality using data collected directly from developing countries.

Accurate excess mortality data is needed for making international comparisons and evaluating policy responses to the pandemic. This paper produces excess mortality estimates for Thailand (as does Wilasang et al.^5^) and analyzes the correlation of excess mortality with restrictions and mobility to contribute to the growing literature on COVID-19 lockdowns. This is the first subnational excess mortality study for Thailand and one of the few studies of subnational excess mortality and lockdowns for a developing country.

This paper uses monthly death data published by an official source in Thailand (Bureau of Registration Administration) disaggregated by province, sex, and age to compile excess mortality statistics for each month between January 2020 and December 2021. To construct excess mortality indicators, counterfactual deaths are estimated in two ways. First is the more common statistical approach of defining long-term average deaths as the average monthly deaths over the previous five years, often with some trend adjustment. However, as Thailand is an aging society, mortality has an upward trend, so this paper also estimates counterfactual deaths using mixed effects modelling to account for trends and heterogeneity at the provincial level. Excess mortality is expressed as a ratio of the unexpected deaths during the period of interest to the long-term average of mortality for a window of the same length. This ratio is the p-score, which is also reported by different age and gender groups to examine mortality risks. An overall age-standardized p-score is compiled to facilitate comparisons to countries with different age distributions.

Results from our model show that from January 2020 to December 2021, Thailand had about 30,000 excess deaths. There is large variation in excess deaths across age groups, gender groups, and provinces. The average p-scores for females and males were similar for the first wave, but males had higher average p-scores in the second and third waves. Those aged 65 and above had significantly higher p-scores in the second wave than younger people. Reductions in mobility are associated with small negative effects on excess mortality, but the estimates are imprecise due to the small sample.

The next section presents a review of related studies on excess mortality due to COVID-19 in other countries. Section "Context: COVID-19 in Thailand" narrates the unfolding of the pandemic in Thailand. In Sections "Results" and "Discussion", we implement a mixed effects model to estimate excess deaths, and present panel regression analysis of the relationship between lockdown stringency, mobility, and excess mortality. Section "Methods" elaborates on the methodology behind the excess mortality estimates and regression analysis results.

## Literature review

Excess mortality due to macro events has been calculated by comparing observed deaths and expected deaths in the absence of the event, with the latter counterfactual typically estimated based on mortality data from a recent period of normalcy. The difference between observed and expected deaths is then attributed to emergent hazards and used as the estimate of excess mortality. This approach was used in early studies on influenza and pneumonia^6^, with the methodology expanded to incorporate stochastic and Bayesian approaches in later decades^7,8^.

Due to the lack of reliably disaggregated mortality data in developing countries, most studies on COVID-19 excess deaths focus on advanced economies. For the United States, Chan et al.^9^, quantify the net impact of the pandemic in the United States and find that there were 375,235 excess deaths in 2020 with 83% attributable to direct effects of the pandemic, and the remaining 17% attributable to indirect effects. Kontis et al.^10^ applied 16 Bayesian models to estimate excess mortality for 40 industrialized countries during the first wave of the pandemic and found that about 1.4 million more people died between February 2020 and February 2021 compared to a counterfactual no-pandemic scenario, with England and Spain experiencing the largest effects. Islam et al.^11^ estimated approximately 1 million excess deaths for 29 high income countries throughout 2020 using an over-dispersed Poisson approach.

Several studies of European countries^12–14^ find that the brunt of excess mortality was borne by males and the elderly. Turning from demographic to geographic variation, Konstantinoudis et al.^15^ show how excess mortality varied across and within five European countries over time and posit that timely lockdowns led to a reduction in community transmissions and excess mortality.

Karlinsky and Kobak^16^ introduce the World Mortality Dataset, which provides more accurate counts of all-cause mortality and gauges excess mortality during the COVD-19 pandemic. Their data account for linear trends and seasonality, and report estimates at a national level for 125 countries and territories. National estimates of excess mortality have been constructed for large countries excluded from the World Mortality Dataset, such as India^17^. But for most developing countries, subnational estimates of excess mortality during this period are unreliable or unavailable. This is especially concerning for highly populated countries that faced long and deep disruptions due to the pandemic. Such analysis is available for some regions (for example, see Lima et al.^18^ on five Latin American countries and Konstantinoudis et al.^15^ for Europe), but no major study has been done for a Southeast Asian country.

## Context: COVID-19 in Thailand

**Figure 1:**
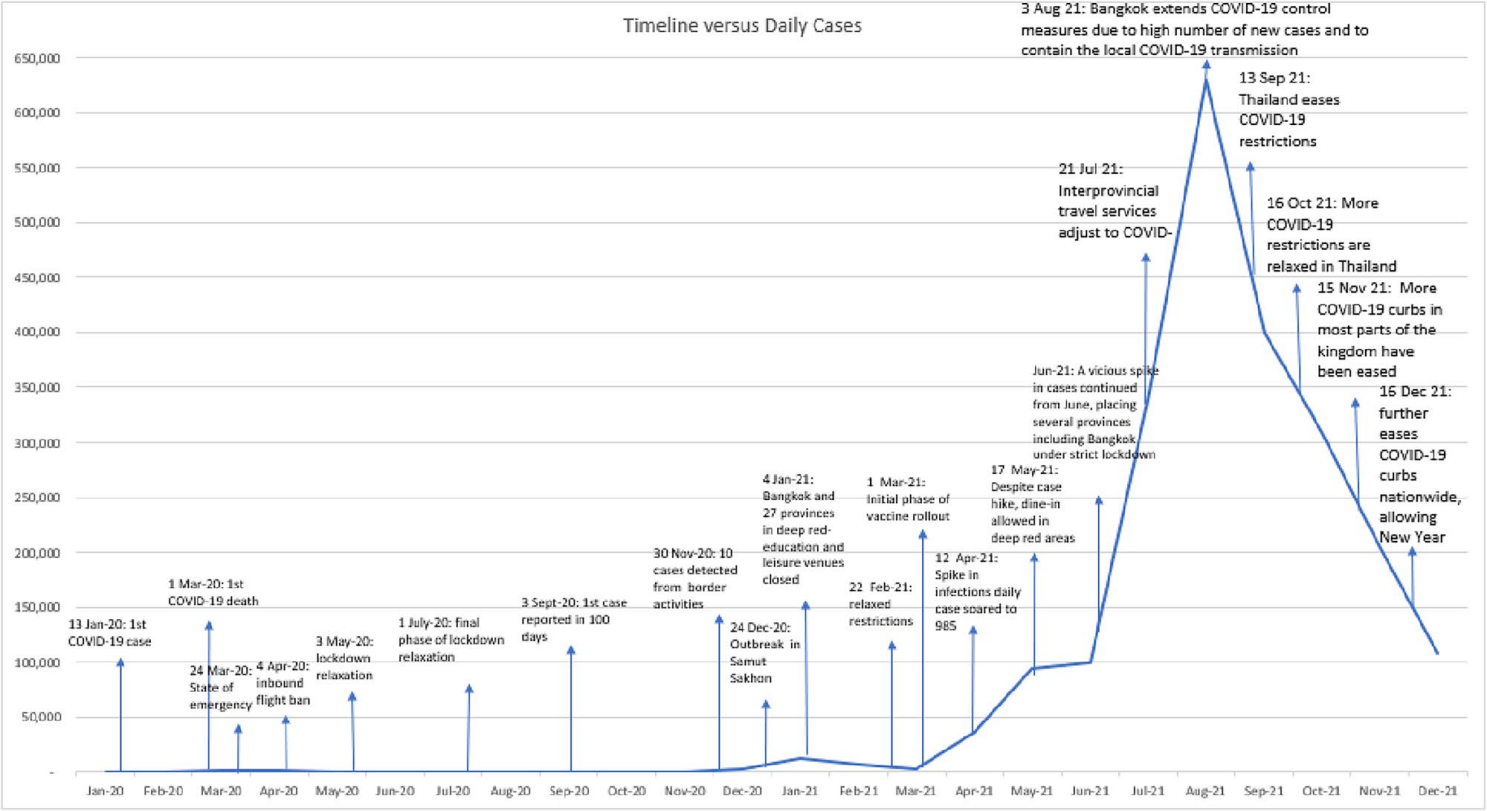
Timeline of COVID-19 cases in Thailand. Source: Authors’ estimates based on data from Our World in Data (https://ourworldindata.org/coronavirus/country/thailand) downloaded on 6 December 2022 and based on articles available in Bangkok Post (https://www.bangkokpost.com/topics/1844044/covid-19), Crisis24, and Tat News. Number of cases is reported on the y-axis.

**Figure 2:**
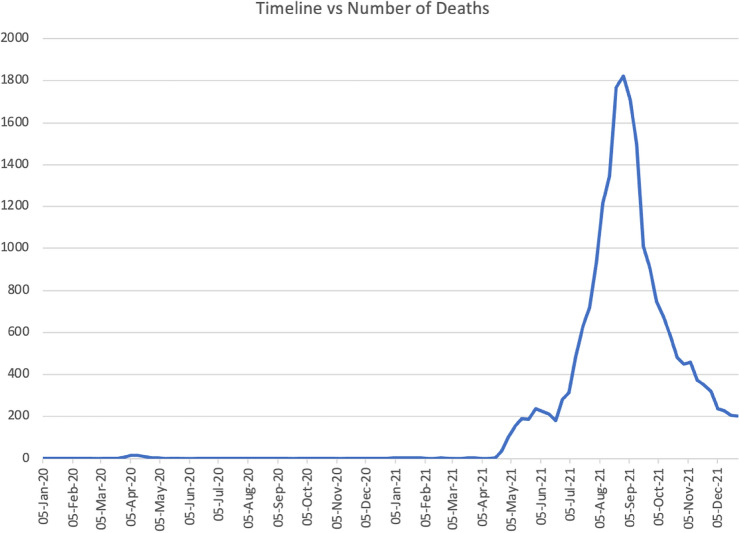
Daily reported COVID-19 deaths in Thailand (January 2020 to December 2021). Source: World Health Organization COVID-19 Dashboard (https://covid19.who.int/WHO-COVID-19-global-data.csv) retrieved on 27 December 2023.

A narrative of how the COVID-19 pandemic unfolded in Thailand is based on newspaper accounts, largely from *Bangkok* *Post*. On 13 January 2020, Thailand became the first country outside of the People’s Republic of China to record a COVID-19 case, marking the soft start of the country’s first COVID-19 wave. By March, Thailand confirmed its first death from COVID-19 in Samut Prakan province. Figure 1 charts the daily confirmed COVID-19 cases and the timeline of policy responses in Thailand across different waves of the pandemic. Figure 2 charts daily reported cases over the study period.

In response to the first outbreak, a state of emergency was declared, effective 26 March 2020, with lockdowns implemented to varying degrees across the country. A nationwide curfew was implemented on 3 April 2020 that was accompanied by measures of strict surveillance and contact tracing by government agencies.

Beginning in May 2020, restrictions were gradually eased as the COVID-19 case count dropped through the month of April. All restrictions, except the ban on international travel, were subsequently removed by July 2020, and schools and entertainment venues were reopened with the resumption of long-distance and tourism train services. Nevertheless, a state of emergency remained in effect until September 2020.

While the government was successful at containing the first outbreak of infections with a nationwide lockdown, it adopted a more targeted approach during the subsequent outbreaks in December 2020 and April 2021. With a new outbreak in Samut Sakhon and Rayong provinces in December 2020, the government adopted stricter surveillance in more affected provinces, zoning provinces into four categories: (1) red zone—maximum control areas as they experienced a large number of infections, (2) orange zone—areas with fewer restrictions and more than 10 reported cases and rising, (3) yellow zone—high surveillance provinces with fewer than 10 reported cases, and (4) green zone—surveillance areas with no reported cases. Some later classification schemes include five colors codes, adding dark red zones, but we group that with red for the purpose of this analysis. The color-coding of a province was changed up to twice in a month, depending on how volatile the situation was. There were restrictions on congregation and closures of recreational establishments as well as schools in the red and orange provinces. In December 2020, travel to various provinces during the New Year was still permitted, except to and from red-zoned Samut Sakhon.

By January 2021, Bangkok and 27 other provinces were under the highest level of restrictions (red zone) with closure of educational institutions and entertainment venues, shopping malls, and supermarkets. Convenience stores could operate only if they adhered to health protocols. During the second wave, daily COVID-19 cases peaked at over 900 on 2 February 2021 but fell to a daily average of 100 by 12 February 2021. From 22 February 2021, cases started to decline, and restrictions were relaxed to a minimum in 54 provinces (green zone), except Samut Sakhon, which continued to be a red zone province until mid-March 2021.

In late March 2021, Thailand had a third outbreak. On 12 April 2021, Thailand recorded a new daily high of 985 reported cases. On 26 April 2021, the country implemented stricter COVID-19 restrictions, especially in Bangkok (the epicenter of the third wave), where schools, entertainment districts, and businesses considered high-risk were closed and restrictions remained in effect until mid-May. The cumulative case count increased seven-fold from 33,000 in April 2021 to 230,000 in July 2021.

Thailand experienced the most consistent and significant increase in daily new infections between July and December 2021, which was the fourth wave of the pandemic. The daily case count peaked at 21,864 in mid-August, and the daily new infections remained as high as 2,600 at the end of the year. Most provinces were categorized as red zones until September 2021. In addition to suspending international travel from Thailand, the government put restrictions on domestic travel from areas with high case counts. But despite a daily case count of over 10,000 in October 2021 (more than double the cases recorded in April 2021), restrictions started to ease in that month, with curfews lifted in dark red zones. Domestic travel restrictions to and from red zones that were in place between July and September 2021 were also lifted in October 2021.

## Results

### Excess mortality estimates

This paper uses generalized linear mixed effects models to estimate excess mortality. Excess mortality is estimated using four different econometric specifications with three outcome variables, separately by age group and gender. Full descriptions of the data and models used are in the Methods section. Table 1 summarizes the average relative errors (ARE) by age group and gender, generated from out-of-sample 2019 predictions of each of the 12 models applied to 2014–2018 data at the province-month level. The prediction errors are averaged across provinces and months to obtain an overall ARE for the age-gender group per model.

**Table 1:** Average relative errors.

Age 0–14	FE poisson			FE negative binomial			ME Poisson random intercept (months)			ME Poisson random intercept (provinces)		
Sex	(a)	(b)	(c)	(a)	(b)	(c)	(a)	(b)	(c)	(a)	(b)	(c)
**Female**	**0.526**	**0.529**	**0.524**	**0.526**	**0.528**	**0.524**	**0.515**	**0.518**	**0.513**	**0.516**	**0.528**	**0.512**
*Wald chi2*	92.19	94.19	66.10	84.86	89.20	62.69	16,274.86	16,294.23	2096.13	92.28	215.84	65.35
*Df*	13	14	13	13	14	13	78	79	78	13	14	13
*Prob* > *chi2*	0.00	0.00	0.00	0.00	0.00	0.00	0.00	0.00	0.00	0.00	0.00	0.00
**Male**	**0.506**	**0.513**	**0.501**	**0.507**	**0.512**	**0.501**	**0.513**	**0.508**	**0.495**	**0.500**	**0.516**	**0.496**
*Wald chi2*	100.61	114.98	79.39	89.23	109.83	73.21	20,664.58	20,723.46	2169.77	100.68	258.20	79.29
*Df*	13	14	13	13	14	13	78	79	79	13	14	13
*Prob* > *chi2*	0.00	0.00	0.00	0.00	0.00	0.00	0.00	0.00	0.00	0.00	0.00	0.00

Age 15–64	FE Poisson			FE negative binomial			ME Poisson random intercept (months)			ME Poisson random intercept (provinces)		
Sex	(a)	(b)	(c)	(a)	(b)	(c)	(a)	(b)	(c)	(a)	(b)	(c)
**Female**	**0.139**	**0.139**	**0.138**	**0.139**	**0.139**	**0.138**	**0.139**	**0.139**	**0.138**	**0.139**	**0.139**	**0.138**
*Wald chi2*	332.28	342.26	332.60	265.55	291.22	283.26	193,344.48	193,332.59	5880.40	332.25	485.87	332.53
*df*	13	14	13	13	14	13	78	79	78	13	14	13
*Prob* > *chi2*	0.00	0.00	0.00	0.00	0.00	0.00	0.00	0.00	0.00	0.00	0.00	0.00
**Male**	**0.099**	**0.099**	**0.099**	**0.098**	**0.098**	**0.099**	**0.098**	**0.098**	**0.099**	**0.098**	**0.099**	**0.099**
*Wald chi2*	1221.98	1242.60	1199.70	822.95	857.22	868.84	397,963.87	399,031.45	17,552.12	1222.02	1373.57	1199.71
*df*	13	14	13	13	14	13	78	79	78	13	14	13
*Prob* > *chi2*	0.00	0.00	0.00	0.00	0.00	0.00	0.00	0.00	0.00	0.00	0.00	0.00

Age 65 +	FE Poisson			FE negative binomial			ME Poisson random intercept (months)			ME Poisson random intercept (provinces)		
Sex	(a)	(b)	(c)	(a)	(b)	(c)	(a)	(b)	(c)	(a)	(b)	(c)
**Female**	**0.112**	**0.112**	**0.112**	**0.112**	**0.112**	**0.111**	**0.110**	**0.110**	**0.110**	**0.110**	**0.110**	**0.110**
*Wald chi2*	2623.86	2626.15	3154.38	1153.41	1163.23	1453.20	394,347.41	394,416.17	6029.57	2623.87	2629.06	3151.34
*df*	13	14	13	13	14	13	78	79	78	13	14	13
*Prob* > *chi2*	0.00	0.00	0.00	0.00	0.00	0.00	0.00	0.00	0.00	0.00	0.00	0.00
**Male**	**0.109**	**0.109**	**0.109**	**0.109**	**0.109**	**0.108**	**0.107**	**0.107**	**0.106**	**0.107**	**0.107**	**0.106**
*Wald chi2*	2569.57	2569.58	2229.37	1297.62	1301.79	1174.25	359,934.13	359,706.76	4486.20	2569.69	2570.68	2228.47
*df*	13	14	13	13	14	13	78	79	78	13	14	13
*Prob* > *chi2*	0.00	0.00	0.00	0.00	0.00	0.00	0.00	0.00	0.00	0.00	0.00	0.00

Specifications a) **Y** is defined as death counts; b) **Y** is defined as death counts with provincial population as a covariate; and c) **Y** is defined as death counts with provincial population as an exposure variable.

Significant values are in [bold].

The 12 models have similar performance in terms of average relative error. Model performance varies across age-gender groups. The models work poorly for the 0–14 years age group, with an average error of about 50 percent, primarily due to the low mortality in this age group. For instance, in 2019, average monthly death rate by province was seven males and five females per 100,000 people in the 0–14 age group. This paper therefore excludes the 0–14 population from the model estimating excess mortality and calculates total deaths as the sum of a model’s estimate for each of the adult and elderly groups. With AREs around 10 percent, the models perform best for the elderly and male adults, who have the highest death counts among the six age-gender cohorts. Model performance results are similar when minimizing mean squared relative prediction errors is set as the objective. A robustness check using 2013–2017 data to predict 2018 deaths yielded similar results. The predicted deaths regressions that form the basis excess mortality estimates can be found in the appendix (Tables A4a, A4b, A5a, and A5b).

For the remainder of the paper, we report results based on the mixed effects Poisson model with province random intercepts, and provincial population as a control (column b in Table 1). This is the preferred specification for several reasons. First, it allows us to account for inherent variability in mortality patterns across geographic areas. Second, it generates estimates with greater precision than the fixed effects negative binomial model due to the stronger parametric assumptions associated with the Poisson model. As Poisson models have potential overdispersion, we also estimate the negative binomial model, which allows closer comparison with other excess mortality papers that cover Thailand during the COVID era^3,5^. The broad trends are similar (Table 2 vs. Table 3). On top of this, column b of Table 1 is preferred because it includes population as a covariate in the most flexible way among the variations in Table 1.

**Table 2:** Mixed effects with province random effects, population as covariate.

Year 2020	J	F	M	A	M	J	J	A	S	O	N	D
Total excess deaths	897.63	− 608.45	1513.19	− 3874.06	− 3239.06	102.06	− 1133.73	− 1177.25	− 1490.31	− 841.76	2316.23	3137.13
LB	− 6476.95	− 7044.18	− 5300.03	− 10,882.7	− 10,084.2	− 6169.1	− 7660.02	− 7903.6	− 8003.52	− 7621.1	− 4247.26	− 3649.02
UB	7035.97	4751.34	7185.98	1962.32	2457.69	5315.88	4294.88	4416.72	3930.42	4800.11	7777.31	8785.70
Total adj. pscore	2.29	0.02	2.26	− 9.99	− 9.38	1.00	− 1.35	− 3.78	− 2.99	− 2.25	3.19	5.84
LB	− 12.41	− 14.34	− 12.38	− 22.89	− 22.37	− 13.48	− 15.49	− 17.57	− 16.89	− 16.25	− 11.61	− 9.36
UB	18.03	15.39	17.93	3.82	4.53	16.49	13.77	10.98	11.88	12.75	19.03	22.12
Female adults	2.99	2.07	0.49	− 6.94	− 8.48	1.54	− 1.67	− 6.35	− 5.25	− 3.35	2.51	4.24
Male adults	1.76	− 1.27	3.31	− 14.19	− 11.61	0.81	0.08	− 1.47	0.04	− 1.17	2.26	6.52
Female elderly	0.96	− 2.59	5.70	− 6.63	− 5.12	0.91	− 5.74	− 3.25	− 5.02	− 1.67	7.92	9.34
Male elderly	2.81	− 2.06	2.91	− 7.36	− 6.31	− 1.46	− 2.95	− 2.12	− 5.01	− 2.54	7.88	7.71

Year 2021	J	F	M	A	M	J	J	A	S	O	N	D
Total excess deaths	− 623.18	− 3597.66	845.15	− 1533.65	1095.54	1598.21	4978.50	9618.51	4610.41	587.66	3313.29	4337.07
LB	− 1271.70	− 4246.17	196.66	− 2182.16	447.05	949.71	4330.01	8970.02	3961.92	− 60.83	2664.80	3688.56
UB	25.35	− 2949.15	1493.64	− 885.14	1744.04	2246.72	5627.00	10,267.00	5258.90	1236.14	3961.78	4985.58
Total adj. pscore	− 4.10	− 7.72	1.90	− 2.27	2.60	3.27	12.80	21.27	12.01	1.84	6.78	7.75
LB	− 20.37	− 23.43	− 15.41	− 18.90	− 14.86	− 14.29	− 6.44	0.59	− 7.08	− 15.51	-11.37	-10.57
UB	14.23	9.95	21.39	16.45	22.24	23.03	34.45	44.52	33.49	21.36	27.20	28.36
Female adults	− 6.03	− 8.49	− 0.13	− 3.42	2.37	2.68	16.78	24.79	14.04	3.94	5.74	7.42
Male adults	− 3.82	− 6.21	4.25	− 0.01	2.90	3.49	9.27	17.85	10.57	− 0.20	7.57	6.99
Female elderly	1.13	− 9.18	1.44	− 6.35	2.77	4.63	10.52	20.08	10.56	1.92	7.30	10.99
Male elderly	0.15	− 10.45	0.97	− 3.77	1.94	3.92	12.11	21.42	9.69	0.88	7.89	10.57

Total adjusted pscore is weighted by age-sex proportions in each province-year. Adult ages are 15–64; elderly are 65 years of age and above.

**Table 3:** Excess Mortality in Thailand using Fixed Effects Negative Binomial model with population as covariate, January 2020 to December 2021.

	W A V E 1												
Indicator	Jan	Feb	Mar	Apr	May	Jun	Jul	Aug	Sep	Oct	Nov		
	2020	2020	2020	2020	2020	2020	2020	2020	2020	2020	2020		
Excess death counts	1,306.31	− 221.54	1929.44	− 3,373.62	− 2,776.24	446.82	− 773.72	− 867.73	− 1,128.75	− 464.02	2,683.15		
Confidence Interval (CI)—Lower Bound (LB)	− 7,211.18	− 7,630	− 5,958.65	− 11,460.7	− 10,732.8	− 6,925.56	− 8,412.55	− 8,755.19	− 8,706.25	− 8,358.43	− 4,952.58		
CI—Upper Bound (UB)	8,006.37	5,609.69	8,131.36	2,988.71	3,471.63	6,222.53	5,217.19	5,314.25	4,819.68	5,732.75	8,678.32		
Age-sex adjusted p-score	2.75	0.50	2.76	− 9.48	− 8.89	1.47	− 0.89	− 3.38	− 2.57	− 1.74	3.70		
CI—LB	− 20.49	− 22.31	− 20.29	− 29.98	− 29.42	− 21.45	− 23.26	− 25.05	− 24.47	− 23.86	− 19.61		
CI—UB	32.09	29.30	31.80	16.45	17.03	30.38	27.31	23.89	25.00	26.13	33.09		
Female adults (ages 15–64)	3.27	2.35	0.77	− 6.63	− 8.17	1.80	− 1.46	− 6.14	− 4.99	− 3.06	2.83		
Male adults (ages 15–64)	2.12	− 0.89	3.73	− 13.84	− 11.27	1.24	0.55	− 1.08	0.36	− 0.67	2.69		
Female elderly (ages 65 +)	2.15	− 1.44	6.78	− 4.99	− 3.72	1.98	− 4.73	− 2.47	− 4.01	− 0.90	8.93		
Male elderly (ages 65 +)	4.22	− 0.52	4.76	− 5.91	− 4.71	− 0.03	− 1.58	− 0.90	− 3.56	− 0.93	9.58		

	WAVE 2				WAVE 3			WAVE 4					
Indicator	Dec	Jan	Feb	Mar	Apr	May	Jun	Jul	Aug	Sep	Oct	Nov	Dec
	2020	2021	2021	2021	2021	2021	2021	2021	2021	2021	2021	2021	2021
Excess death counts	3,534.46	− 537.08	− 3,377.34	1,076.46	− 1,184.20	1,466.30	1,977.88	5,968.30	10,729.58	5,057.04	930.84	3,558.92	4,466.05
CI- LB	− 4,304.18	− 5,681.49	− 7,890.65	− 3,870.16	− 5,923.81	− 3,300.52	− 2,553.98	1,278.82	5,676.08	381.45	− 4,009.92	− 1,352.56	− 593.06
CI—UB	9,697.65	3,967.81	575.52	5,408.75	2,966.24	5,640.20	5,944.66	10,072.80	15,153.51	9,150.89	5,257.51	7,860.30	8,896.95
Age-sex adj. p-score	6.37	− 3.98	− 7.63	1.92	− 1.96	3.14	3.73	15.25	23.57	12.64	2.20	6.78	7.68
CI—LB	− 17.54	− 14.78	− 18.03	− 9.53	− 13.00	− 8.46	− 7.94	2.24	9.63	− 0.04	− 9.30	− 5.21	− 4.42
CI—UB	36.51	7.91	3.81	14.50	10.19	15.89	16.56	29.56	38.88	26.58	14.85	19.97	20.99
Female adults	4.48	− 6.02	− 8.57	− 0.26	− 3.12	3.16	3.12	19.72	27.53	14.78	4.43	5.46	7.11
Male adults	7.08	− 3.61	− 6.17	4.09	− 0.01	2.94	3.64	11.04	19.21	10.73	− 0.25	7.52	6.95
Female elderly	10.50	1.25	− 8.67	2.13	− 5.00	3.82	5.97	13.27	23.46	11.67	2.86	7.88	11.46
Male elderly	9.35	0.44	− 9.39	2.17	− 2.73	3.44	5.49	15.18	25.39	11.97	2.32	9.40	11.25

Source: Authors’ estimates for population aged 15 and above.

**Figure 3:**
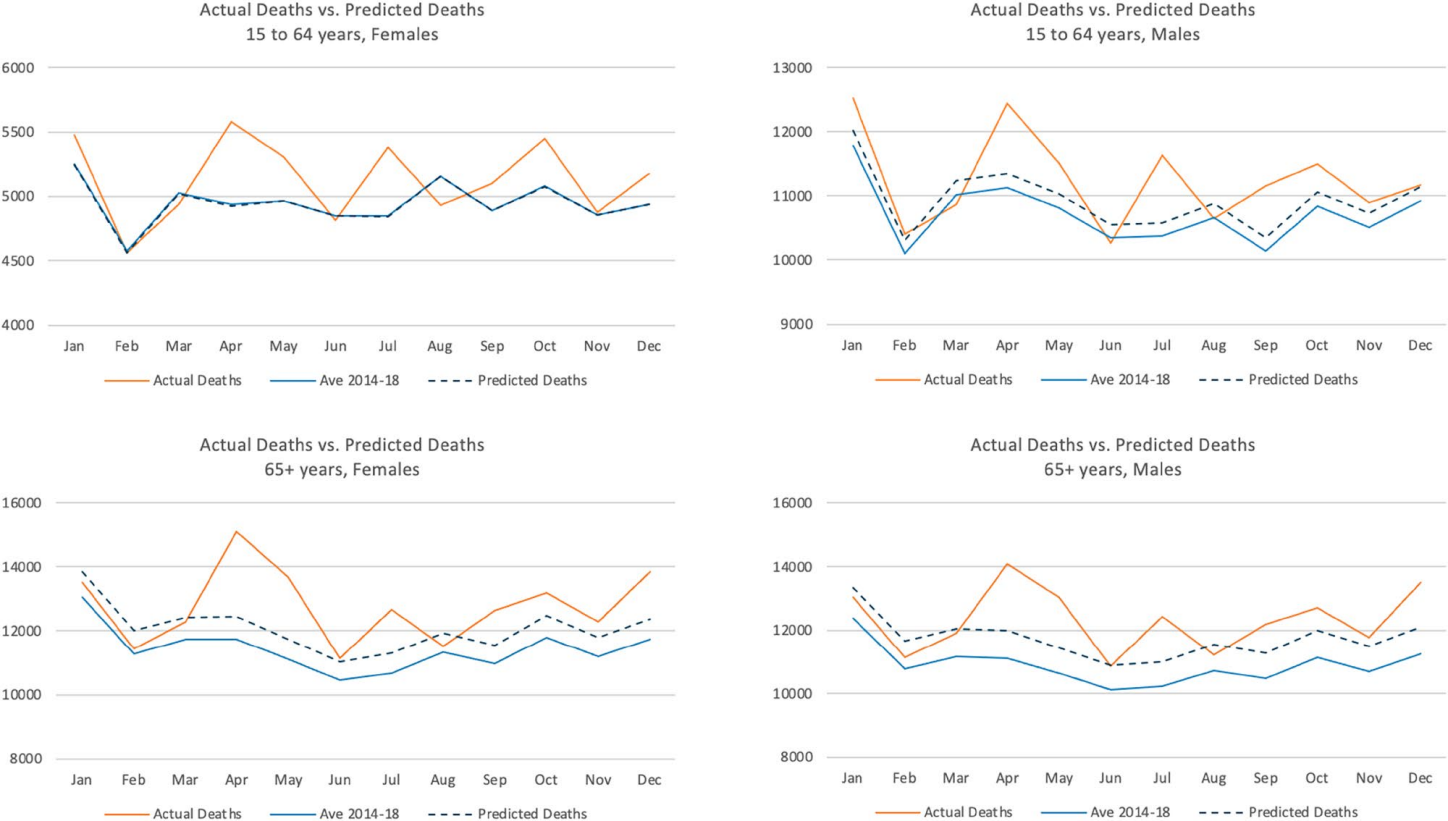
Five-year average versus predicted Deaths in 2019. Source: Authors’ estimates based on mixed effects model.

Figure 3 indicates the difference between our preferred model’s predicted deaths and the average deaths in the preceding five years by age-gender group. Predicted deaths are greater than the five-year average in all cases, which is expected because model predictions account for the trends in aging and rising deaths. Figure 3 also charts the actual deaths recorded in 2019. Unsurprisingly, excess mortality would have been overestimated if simple, unadjusted five-year averages were used as counterfactual deaths.

Although Thailand reported its first COVID-19 case in January 2020, it only recorded its first death from the disease two months later (Fig. 1). But Tables 2 and 3, which applies the model to predict counterfactual 2020 and 2021 deaths and estimate excess mortality for January 2020-December 2021, indicate that there was significant excess mortality even in January 2020. This suggests that the deaths at the start of the pandemic could have been underestimated, which is unsurprising since testing was infrequent and health protocols had not been established early in the pandemic.

While reports suggest that Thailand’s second wave commenced in early December 2020, our results imply that it may have started in November 2020 when excess mortality figures turned positive, with 2,300 deaths more than expected (Table 2). As shown in Fig. 1, a cluster of new infections developed in provincial areas as early as November 2020. A larger outbreak in December 2020, centered in Samut Sakhon, corresponded with excess deaths of about 3,100. Excess deaths turned negative in January 2021 (-600 excess deaths) as the country entered the strictest lockdown in the second wave. During this month, Bangkok and 27 other provinces were categorized as “deep red” zones, with the most stringent containment measures, resulting in the closure of schools, entertainment, and recreational venues.

Excess mortality at the start of the second wave was higher than in the first. The pattern was most pronounced for the elderly, who experienced consistently higher mortality (by 7–9 percent) than adults (2–7 percent).

Thailand entered the third wave of the COVID-19 pandemic in late March 2021, with a fresh outbreak in Bangkok clustered in a string of entertainment complexes. About 845 excess deaths are estimated for March 2021. The excess death count turned negative again in April 2021, when the stricter movement restrictions imposed in Bangkok during this time.

Although the estimated p-scores were relatively high for older age groups in June, the observed excess deaths in these groups were lower than expected when COVID-19 restrictions began in April 2021 (a decline of 3–6 percent) Estimated p-scores by gender and age group turned positive in May and June 2021 when COVID-19 restrictions started to relax in the second half of May.

Excess deaths peaked again during August 2021, with a surge in infections due to the delta variant. During this month, all groups had elevated p-scores, although for a short period, female adults had disproportionately more excess deaths. Concurrent with continued reduction in lockdown strictness through August to December 2021, the excess mortality, while lower than August 2021, continued to be higher in that fourth wave than in the earlier waves of the pandemic.

**Figure 4:**
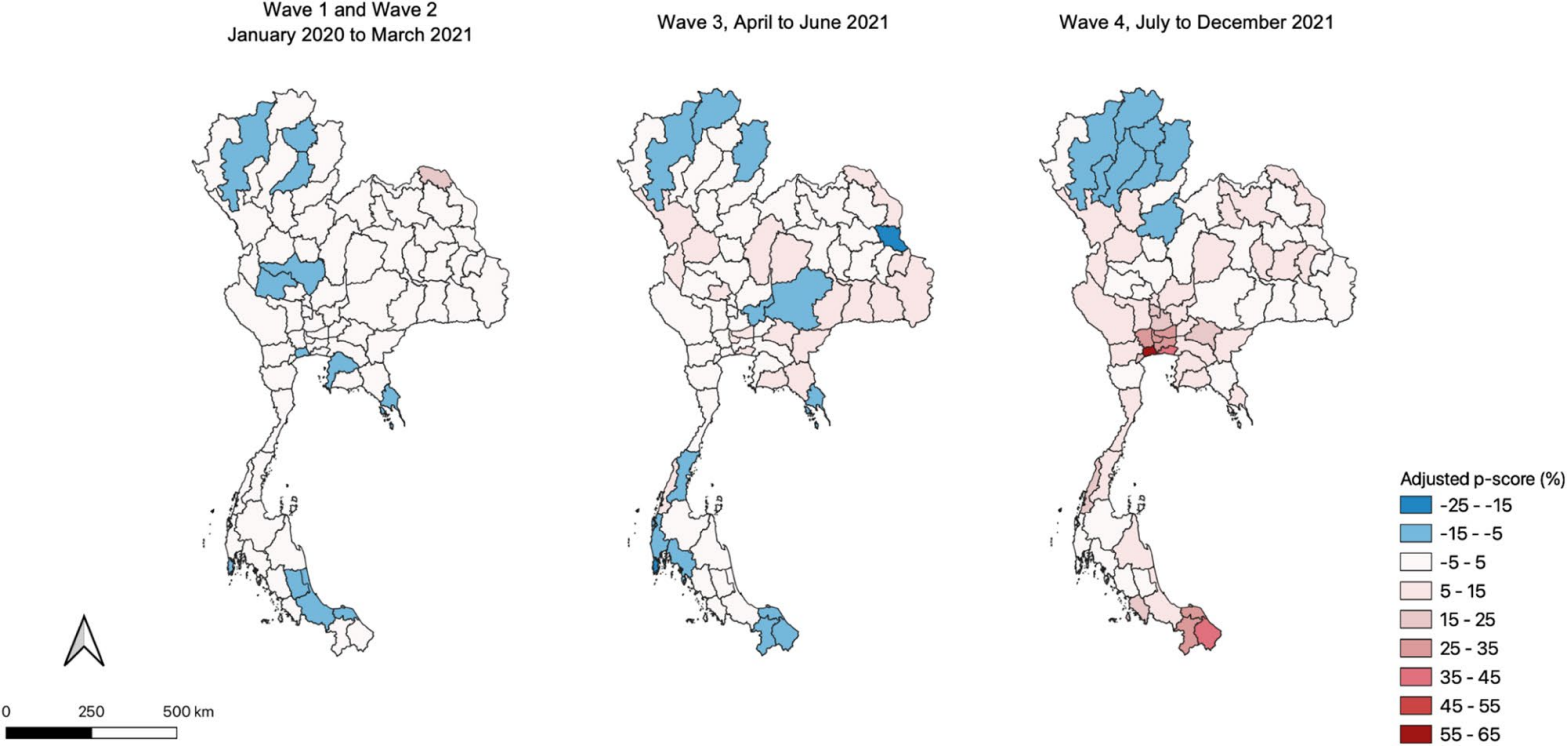
Province-level excess mortality (adjusted p-score) by pandemic wave. QGIS was used to generate these maps. It is a free and open-source software for GIS and remote sensing applications that supports viewing, editing, and analysis of geospatial data.

Figure 4 illustrates how excess deaths in Thailand were driven by provinces in the vicinity of Bangkok, largely during the fourth wave of the pandemic. The maps also indicate that provinces in the northwest largely escaped the pandemic and apparently benefitted from the behavioral caution that came with restrictions, with fewer than expected deaths throughout the period.

### Were lockdown measures successful in restricting mobility?

Stringency measures were negatively correlated with individuals’ movements, as measured by a mobility index constructed using Facebook data. This measurement, *StayPut*, captures the increase in the monthly average share of individuals who stayed within the 0.6-square km throughout the day, and is inverse to individual movements—(see appendix for details). Table A1 shows that being red-coded is significantly positively related with the share of a province’s Facebook users staying in or near their homes. While being in the red zone was associated with a 6 percent higher share of individuals restricting mobility (compared to an average of 19 percent staying near homes in the green zones), *StayPut* mobility index is no different for yellow versus green zones.

**Figure 5:**
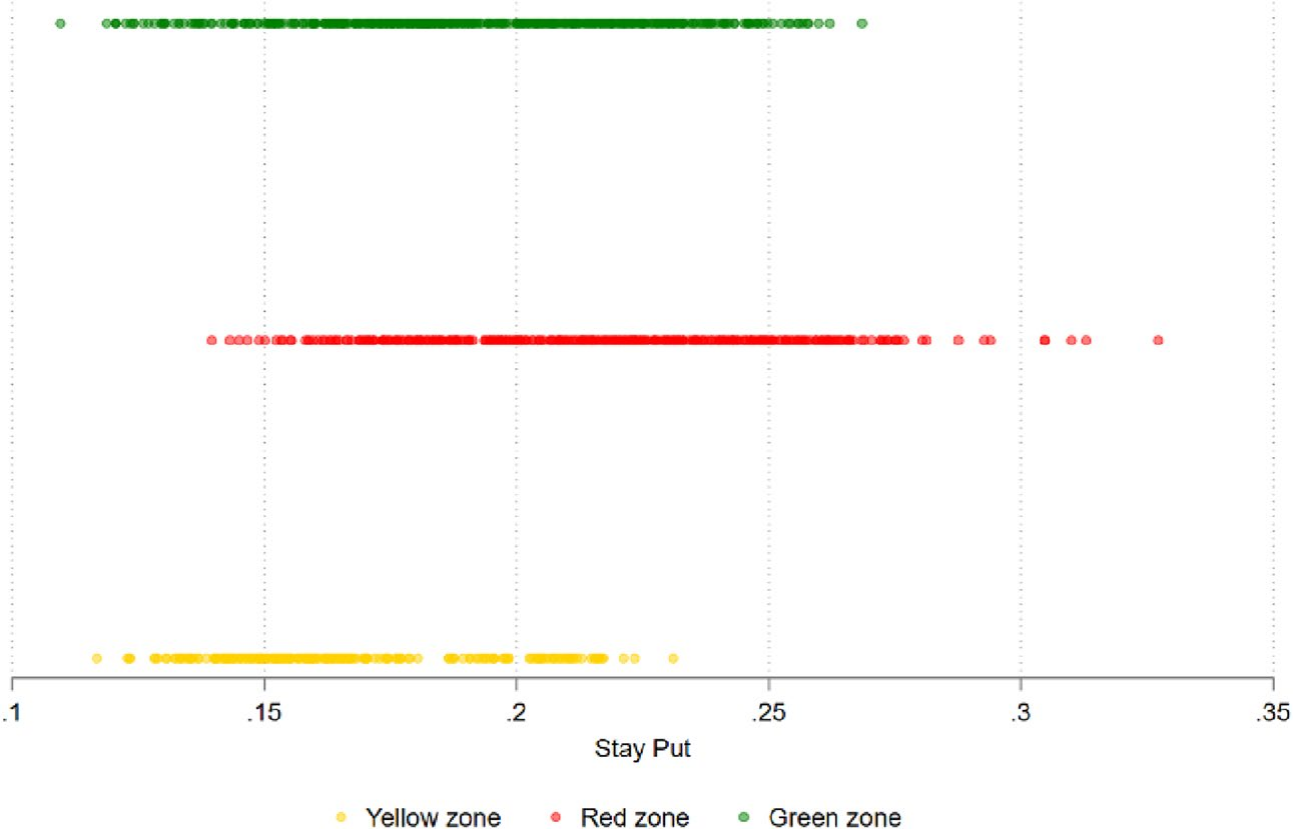
Lockdown stringency and mobility. *Note*: The sample is restricted between December 2020 and December 2021, for when the color-coded zoning is available. The red zone combined dark red and red government defined color zones, yellow zone combines orange and yellow and green zone combines green and blue. *Stay Put* is the Facebook mobility index (share of users staying in or close to home over a 24-h period), whose higher value indicates lower mobility.

However, there was substantial heterogeneity in the effectiveness of color coding in reducing movements during the two years of the pandemic. As shown in Fig. 5, there is a large overlap in the mobility index between green, yellow, and red zones. Moreover, there is a wide variation in the mobility index even within red zones, which implies that the color-coded zoning was applied unevenly across time and space. This is consistent with our earlier discussion on easing restrictions in red zones towards the latter half of the study period (see Fig. 1 for details). Additionally, we notice high *StayPut* in some green zones, which may reflect people’s preference for staying in, thereby, resulting in a lower-case count, even though there were few or no mobility restrictions.

To study whether lockdowns were effective in reducing excess mortality, we use both the government-defined color-coding, implemented between December 2020 and December 2021, as well as the Facebook mobility index *StayPut*, available between March 2020 and December 2021, that directly captures the movements that lockdowns were intended to restrict. *StayPut* has the advantages of a longer data series and higher variation as a dependent variable.

### Were lockdown measures and restricted mobility successful in reducing mortality?

Given the transmission duration, mobility restrictions in the current month are expected to impact the following month’s excess mortality. Table A2 reports results from an autoregressive distributed lag model. The lag between the timing of mobility restrictions and its effect on containing infections (and consequently deaths) is exploited to estimate the (lagged) effect of lockdown-induced mobility restrictions on excess mortality (as estimated by the mixed effects Poisson model—results are virtually identical based on excess mortality estimated by the negative binomial model) in the following period.

Results suggest a negative contemporaneous relation between movement and excess mortality, with provinces with lower mobility experiencing higher excess deaths. This is unsurprising since restrictions on mobility were adjusted in response to provincial case counts. However, the lagged effect of mobility is positive in Table A2 (but insignificant due to the small sample, almost 100 fixed effects, and clustered standard errors), suggesting that lower mobility in the past month may have reduced excess mortality in the following month. Table A3 reiterates these findings across the four waves of the pandemic. Lower mobility in previous months is associated with lower excess mortality, specifically in waves 1 and 3. These results are suggestive and indicate that trends in the data point to restrictions reducing excess deaths. However, we refrain from causal interpretations.

**Figure 6:**
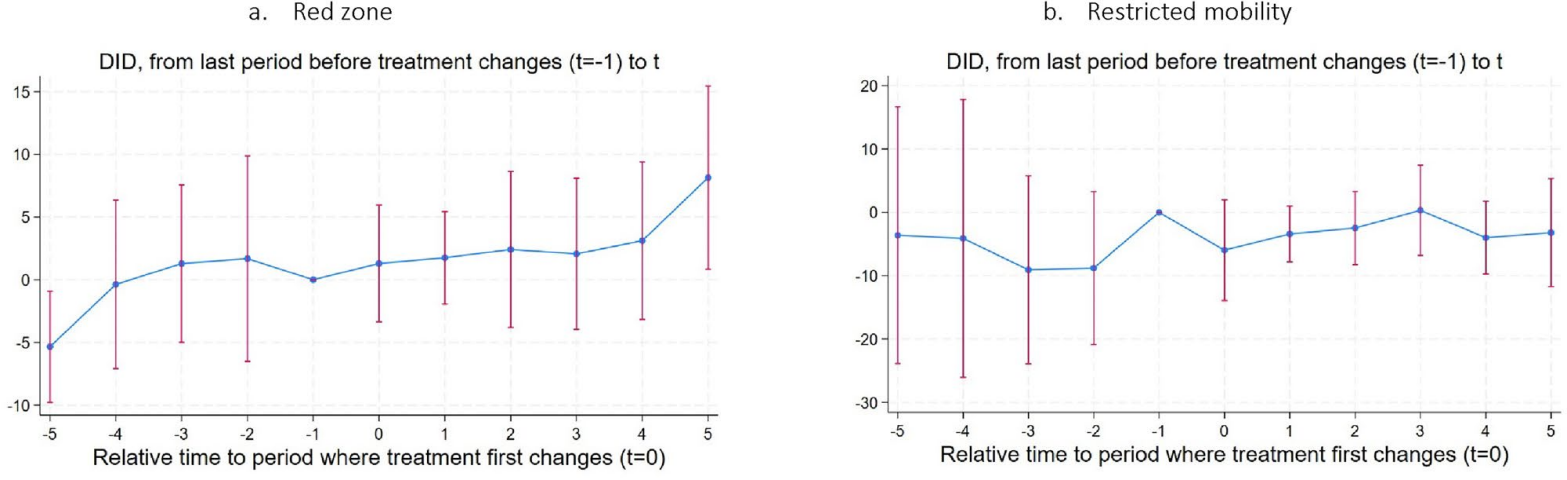
Difference-in differences results using de Chaisemartin and d’Haultfoeuille^19^** estimator.** *Note*: These results are plotted using *did_multiplgt* command in STATA. Treatment in panel (**a**) is the indicator variable which is 1 for when the province turned red and in panel (**b**) is the indicator variable that takes value 1 when share of individuals staying within 0.6 sq-km in a day was above 21.5%, the 75^th^ value in the *StayPut* distribution over the entire study period. For 77 provinces, the sample is restricted between December 2020-December 2021 in panel (**a**), for when color-codes are available, and the study sample is for March 2020 to December 2021 in panel (**b**). Each coefficient on x-axis is the effect #k (greater than 0) is the DID estimator for the effect of having switched into red status for the first k months ago. The effect #k (less than 0) are the placebo effects. Standard errors are computed over 50 bootstrapped samples and clustered at the province level. See de Chaisemartin and d’Haultfoeuille^19^ for details on the estimation strategy. 95% confidence intervals depicted.

The simultaneity of color-coding and COVID-19 case counts means that any regression estimates will suffer from an endogeneity problem. However, recent econometric advances in difference-in-differences methodology can improve the quality of correlational analysis. Figure 6 presents results of the event study from Eq. (3), a difference-in-differences estimator proposed in de Chaisemartin and d’Haultfoeuille^19^, which allows for switching treatment status to study the effect on excess mortality of two binary treatment variables: (i) the first-time switchers into red zone status (Fig. 6a); and (ii) an indicator for high restricted mobility that takes value 1 if the Facebook mobility index in a given month was above 21.5%, i.e. the 75^th^ percentile value of the *StayPut* index distribution through the study period (Fig. 6b). While the former measure captures the effect of the official intervention, the latter is a direct measure of precautions and adherence followed by individuals, that the lockdown policy intended to nudge. We study the effect of the first-time switches into treatment and show dynamic effects and placebo effects (see Methods for details).

Figure 6a shows that lockdowns do not seem to reduce excess mortality (again estimated by the mixed effects Poisson model, with virtually identical results based the negative binomial model), consistent with large overlaps in mobility index between yellow and red zones. Rather, the effect is close to zero and statistically insignificant. However, it is possible that the heterogeneity in lockdown efficacy uncovered in Table A3 is muddling the picture. In Fig. 6b, the same difference-in-differences estimator is applied, but with restricted mobility itself as the treatment variable. This shows that, when analyzing mobility directly, lockdowns are associated with lower excess mortality, but again the confidence intervals are too large for conclusiveness. These results are insensitive to inclusion of additional controls such as province monthly temperature.

## Discussion

Estimates of excess mortality show that the toll of the COVID-19 pandemic on Thailand between January 2020 and December 2021 was 20,831 based on the Poisson mixed effects model (30,427 based on the negative binomial model). This is approximately 19% (4%) lower than the point estimate of Wilasang et al.^5^ (after restricting our estimates to match their study period), with a similar estimation of negative excess mortality in April 2021 followed by ever rising excess mortality through October 2021. This paper’s estimate of excess mortality is 18% (3%) lower than the estimate of Msemburi et al.^3^ for all of 2021. For 2020, only the mixed effects model matches Msemburi et al.’s estimate of negative excess mortality in 2020. With similarities in methodology and age- and gender-adjustments, our estimates corroborate the other papers’ negative binomial model-based estimates of 2021 excess mortality in Thailand using province-level mortality data for the first time. Our estimate accounts for unreported COVID-19 related deaths, and indirectly caused deaths (due to health system stress, income shocks arising from lockdowns, and other pandemic-related complications).

As excess mortality is a net outcome, the figure also includes any lives saved indirectly by containment policies. This turns out to be substantial for Thailand (with excess mortality negative in 10 out of the 24 months studied), and we hypothesize that this is due to a significant aversion of traffic related deaths (which were responsible for 20,000 deaths per year, pre-COVID). Indeed, road deaths were about 6,000 deaths lower in 2020 and 2021 than in 2018 and 2019. Road deaths have since edged back toward pre-pandemic levels with the corresponding reduction in mobility restrictions^20^.

During periods of high excess mortality, it was the elderly that had the highest mortality risk. Conversely, during periods of containment when excess mortality was negative, adults saw a larger drop in mortality risk than the elderly. This suggests that while the disease was more likely to be fatal for the elderly, control measures had the unintended benefit of reducing adult deaths due to other causes, such as traffic accidents.

Mortality risk varied significantly across the provinces, likely due to the different health infrastructure and poverty levels. We find that stricter controls at the provincial level correlated with lower mobility. While endogeneity issues and data limitations preclude a strong causal conclusion, regression analysis using panel methods show that mobility reductions were associated with lower excess mortality. However, it appears that the lockdowns were applied too unevenly across time and space (or had waning effectiveness due to lockdown fatigue) to show a direct positive correlation with excess mortality.

## Methods

### Excess mortality estimation

The standard approach to estimating excess deaths is to proxy long-term average deaths using data from the previous five years and compare that against the most recent year’s actual deaths. However, this approach does not take into consideration demographic shifts, as well as long- and short-term trends in mortality, which are particularly significant for some age groups^21^. Thailand is an aging society (see Figure A2), so there is a need to account for the upward trend in death counts. We use a variety of models to predict counterfactual deaths, or deaths in the absence of COVID-19.

Excess mortality is then defined as the difference between actual deaths during the pandemic and counterfactual deaths. Three outcome variables are defined (Tables 2 and 3): (i) excess deaths; (ii) the p-score, which is the ratio of excess deaths to projected deaths, and (iii) the age-sex-adjusted p-score, which is derived as a weighted average of the age-sex-specific p-scores, where the weights are the proportions of persons in the corresponding age-sex groups.

The class of models is based on the generalized linear mixed-effects model wherein:

${y}_{it}|\gamma$ has distribution in the exponential family

1 $$E\left[{y}_{it}|\gamma \right]={\mu }_{it}$$

$$g({\mu }_{it})={X}_{it}\beta +{Z}_{it}\gamma$$

***y*** is the dependent variable, **X** is the matrix of fixed effects, **β** is a vector of parameters for the fixed effects, ***Z*** is a matrix of all random effects, and ${\varvec{\gamma}}$ is the parameter vector for the random effects.

The response variable y is modeled using different distributions, and a linear model is assumed for the expected value of ***y***, that is, the link function, denoted here by **g**(.) is linear.

Fixed Effects (β):

- **Temperature in month (t) in province (i)**: This continuous variable characterizes the impact of temperature on mortality.
- **Population in month (t) in province (i)**: Another continuous variable capturing the size of the population in province (i) during month (t).
- **Monthly indicators:** We incorporated a set of categorical indicators to capture the inherent seasonality in mortality rates across different months.
- **Month-year trend:** The linear trend term (trend) allows us to model the overall month-year variations in excess mortality.

Random Effects (γ):

- **Province:** A random intercept for each province to account for the variation in mortality patterns across geographic areas.
- **Month:** A random slope for each month to capture the varying rates of change in excess mortality over time.

A range of models are estimated to test for robustness of results. The four econometric specifications are: (1) Poisson link function with fixed effects only; (2) negative binomial link function with fixed effects only; (3) mixed effects Poisson link with random intercepts for provinces; (4) mixed effects Poisson link with random intercepts for months. The main difference between Poisson and negative binomial link is that the former imposes that the mean of Y be equal to its variance, while the latter accounts for variability exceeding the mean, or “overdispersion”.

Each specification is used with variations to account for heterogenous population trends across provinces: (i) death count as outcome, (ii) death count as outcome with provincial population as a covariate, and (iii) death count as outcome with provincial population as an exposure variable (i.e., restricting the coefficient on provincial population to equal 1). As trends are likely to differ by age and gender, 12 models are applied to male and female sub-populations separately in three age-cohorts: young (0–14 years), adult (15–64) and elderly (65 and above).

### Auto-regressive distributed lag (ARDL) model

Next, to understand how COVID-induced movement restrictions affected excess mortality, a linear autoregressive distributed lag (ARDL) model with one lag for the outcome and two lags for the explanatory variable is used:

2 $${y}_{\left\{it\right\}}={\beta }_{1}{ y}_{\left\{it-1\right\}}+ {\beta }_{2}StayPu{t}_{\left\{it\right\}}+ {\beta }_{3}StayPu{t}_{\left\{it-1\right\}}+{p}_{i}+{t}_{t}+{v}_{\left\{it\right\}}$$

Here, $i$ and $t$ denote province and month, respectively, $y$ is one of the three measures of excess mortality: the number of excess deaths, p-scores, or p-scores adjusted for age and gender (all based on mixed effects Poisson model estimates), $StayPut$ is a proxy variable capturing mobility restrictions, a monthly aggregate at the province level of the fraction of Facebook users who stayed within one 0.6-square km area throughout the day. Using this specification, we exploit the variation in the timing of imposition of COVID-related stringency measures and the lag with which it will contain infection (and consequently deaths) to estimate the (lagged) effect of lockdown-induced mobility restrictions on excess mortality in the following period. Thus, ${\beta }_{2}$ captures the contemporaneous relationship between mobility restrictions and excess deaths, and ${\beta }_{3}$ measures the effect of lagged mobility restrictions on excess mortality. Standard errors are clustered at the province level and month-year and province fixed effects are included to eliminate any omitted variable bias due to unobserved variables that evolve over time or are constant at the province level.

In another specification, the lagged monthly value of $StayPut$ is interacted with an indicator variable for wave 1, 2, 3, and 4, respectively, to understand if restrictions had a differential effect during each of these waves.

### Difference-in-Differences estimator proposed in de Chaisemartin and d'Haultfoeuille^19^

The lockdowns were staggered in Thailand, and provinces moved in and out of red-zone status (treatment) over the period when the color-coded policy was implemented to restrict mobility. Since lockdowns were imposed in areas where case counts (and possible mortality) were high, a simple regression model will be biased and not allow for a causal interpretation. To study whether the lockdown policy and the restricted movements had a role in reducing excess deaths, we implement a difference-in-differences (DiD) methodology. A standard two-way fixed effects estimator to recover the DiD coefficients may be biased since the implementation of policy was staggered and treatment switched on only if infection rates were high, implying that there could be heterogeneous treatment effects^19,22–24^.

Thus, we implement a staggered DiD estimation strategy to understand the effects of lockdown measures on excess deaths as proposed in de Chaisemartin and d'Haultfoeuille^23^, which unlike the standard 2 × 2 DiD framework is robust to endogeneity concerns arising due to staggered treatment implementation and heterogenous treatment effects. This estimator allows for a situation in which the provinces may move in and out of treatment. We use this estimation strategy to study the effect of two binary treatments on excess deaths based on the mixed effects model: (i) red-zone status, and (ii) a high mobility index (*Stay* *Put*), which is an indicator variable that takes the value 1 for a province in a month if *StayPut* is above 21.5 percent, the 75th percentile of the *Stay* *Put* values observed during the entire study period. While the former intends to capture the effect of the lockdown policy, the latter tries to capture the effect of adherence of restrictions that the policy intended to nudge.

The de Chaisemartin and d'Haultfoeuille^23^ estimator is estimated as a dynamic two-way fixed effect estimator within an event study framework to recover the effect of first switches into treatment (into red status or mobility index above 75^th^ percentile) on excess deaths:

3 $${y}_{\left\{it\right\}}={p}_{i}+{m}_{t}+ \sum_{k=-L}^{-1}{\gamma }_{k}1\{{T}_{it}=k\}++ \sum_{k=1}^{U}{\pi }_{k}1\left\{{T}_{it}=k\right\}+{u}_{it}$$

where ${p}_{i},{m}_{t}$ are the province and month-year fixed effects, and $i, t$ denote province and month-year, respectively. The outcome variable is the p-score of excess deaths adjusted for age and gender. ${T}_{it}$ is the treatment variable which is 1 for the month $k$, when province turned red for the first time or when mobility index exceeded the value at the 75th percentile for the first time. The coefficients ${\gamma }_{k}$ correspond to time leading up to the treatment (placebo effects) and measure the effect on the adjusted p-score of excess deaths k period before treatment. The coefficients ${\pi }_{k}$ correspond to the dynamic effects in the time periods following the treatment and measures the effect of having experienced lockdowns for the first-time k periods ago. Specifically, ${\pi }_{k}$ estimates the kth dynamic effect, by comparing the outcomes evolution of *first-time switchers'* and *not-yet switchers'*, from the last period before first-time switchers' treatment status changes (k − 1 period) to the kth period after that change. ${\pi }_{0}$ indicates the instantaneous effect of lockdown on the province’s excess mortality in the month the province goes into red status compared to the month before treatment. We include five dynamic effects and four pre-treatment periods in this specification. Month and province fixed effects are also included.

To the extent that infection rates may affect both the excess mortality and mobility, we posit that province and month fixed effects help rid omitted variable bias in the absence of sub-national data on case counts during the study period. By focusing on the outcome evolution of first-time switchers into treatment in an attempt to establish casual relations, we are limited in using the later switches in a setting where treatment switches were frequent. While causal estimates of the impact of lockdowns cannot be estimated in the absence of exogenous variation in color coding, these estimates are, therefore, improvements on purely correlational estimates of the relationship between lockdowns and mortality.

### Data

To implement these models, data from Thailand’s vital registration system are used. Among countries in Asia, only a few record mortality data at more frequent intervals than annual. Most do not have death data disaggregated by province, gender, and age. Thailand is a notable exception as it has an excellent vital registration system, in which deaths must be registered within 24 h. Since 1991, the Bureau of Registration Administration (BORA) has been the central agency mandated for the civil registration of births and deaths. It runs a dense network of offices at different administrative levels: local, district, municipality, and province. Death registration involves notifying authorities, which will validate deaths before registration. Suspicious deaths are referred to local police for further investigation^25,26^.

Administrative death data used in this paper are, thus, reasonably frequent and of good quality. The availability of mortality data at the provincial level also allows for estimating within-country variations, without having to rely on cross-country models that would hinge on the assumption that Thailand has similar death patterns to developed countries. As population data are only available on an annual basis, we use the annual number of persons in the age-gender group as proxies for the month (i.e., we assume no month-to-month population change). Moreover, as temperature correlates with deaths^27–29^, temperature levels are included as a covariate. Table 4 summarizes the data used in this paper and the sources.

**Table 4:** Data sources.

Variable Name	Source	Period
Deaths (monthly), by age, gender, and province	Bureau of Registration Administration https://stat.bora.dopa.go.th/	January 2009–December 2021
Population (annual), by age, gender, and province	National Statistics Office of Thailand http://www.nso.go.th/	2014–2021
Temperature (monthly), by province*	Copernicus Climate Change Service Climate Data Store https://cds.climate.copernicus.eu/ cdsapp#!/home	January 2009–December 2021
Color-coded (monthly) stringency measures	Centre for COVID-19 Situation Administration (CCSA)	December 2020-December 2021
StayPut (daily)	Humanitarian Data Exchange https://data.humdata.org/	February 2020–December 2021

Death data are accessed on 17 May 2022; population data on 6 May 2022; and temperature data on 19 May 2022.

Temperature is used as a covariate in excess deaths estimation since temperature is known to correlate with deaths^27–29^; mobility data for January–February 2020 are not available. February 2020 is defined as the pre-pandemic level and is used as the baseline.

Figure A2 indicates that Thailand’s population has high proportions of middle-aged and elderly population, and relatively few young people. Women make up the majority of Thailand’s elderly population (45 and above). Improvements in the healthcare system as well as the government’s success in birth control campaigns have led to Thailand’s rapidly aging population. In Thailand, the total fertility rate declined from 6.4 children per woman in the 1950s to about 1.5 in 2019 and is expected to further decline during the next 20 years. Over the same period, life expectancy at birth increased from 54 to 77 years between 1960 and 2019 and is expected to increase further to 79 years by 2050.

**Figure 7:**
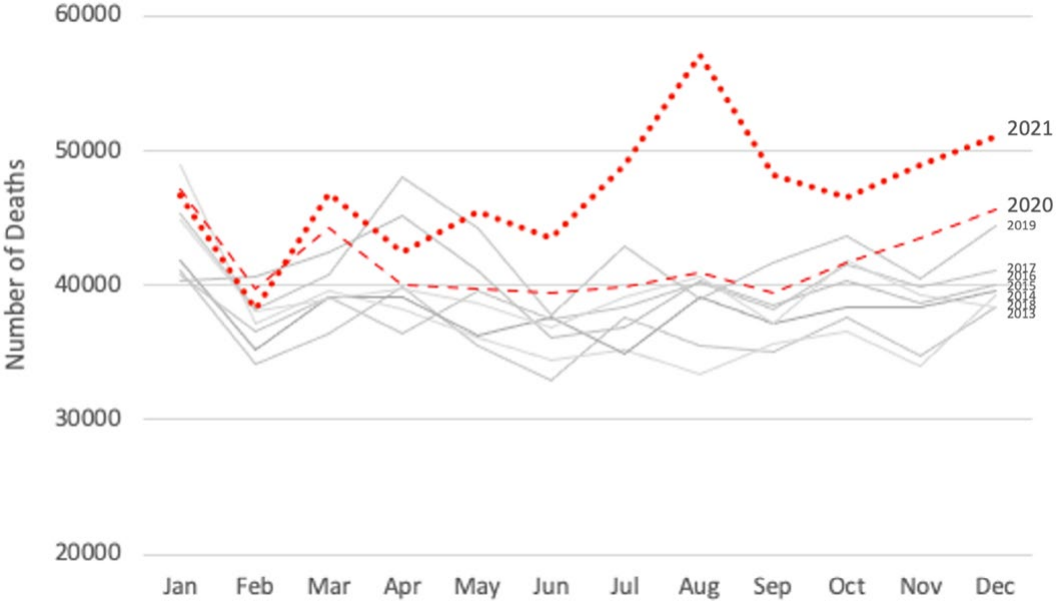
Mortality trends in Thailand (2013–2021). Source: Authors’ estimates based on BORA deaths data.

In March 2020, at the height of the pandemic during the first wave, mortality was markedly higher than the average across the previous five years (Fig. 7). Overall, monthly deaths from January 2020 to June 2021 are higher than their respective months in the years before the pandemic, except in April and May 2020 when a nationwide night-time curfew was imposed.

Thailand’s peak mortality for any given year is observed in the months of January, April, and December, which may be attributed to the two big holiday seasons during those months: the New Year in late December to early January, and the Songkran festival in April. According to WHO’s 2018 Global status report on road safety^30^, Thailand has one of the highest road traffic death rates in the world, and the issue is at its worst during those holiday periods.

**Figure 8:**
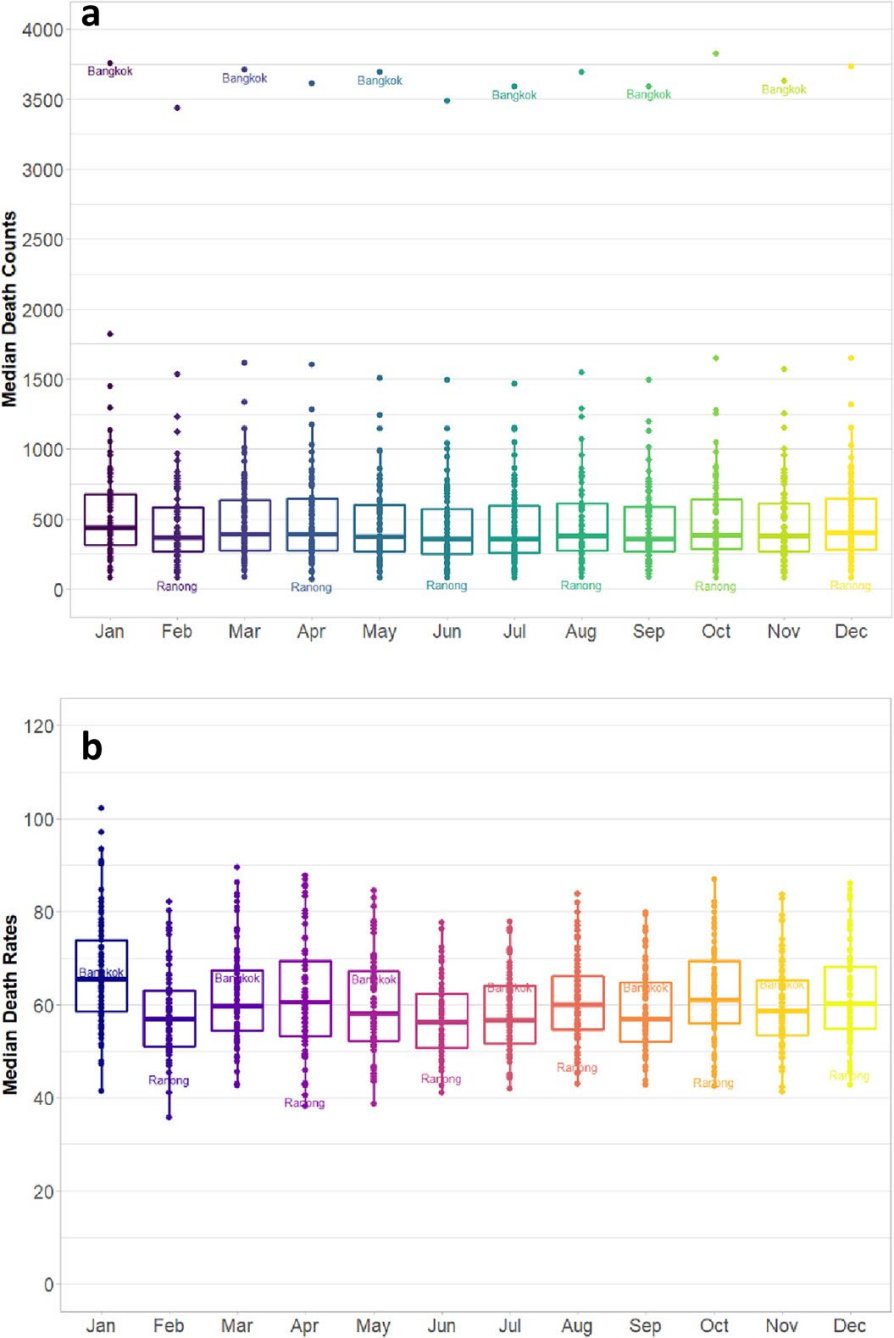
(**a**) Median Deaths across 77 Provinces of Thailand (2014–2019). (**b**) Median Death rates across 77 THA Provinces (2014–2019). Source: Authors’ estimates based on BORA death data.

Death counts vary significantly across provinces (Fig. 8a). Bangkok and Ranong are the most and least populous provinces and have the highest and lowest number of deaths, respectively. For the 77 provinces, the median number of deaths is about 400 for all months, with the distribution skewed to the right. Figure 8b shows the median death rate of around 60 per 100,000 population each month. The small death counts and rates at the provincial level imply that minor discrepancies in estimates may result in relatively large estimation errors and the wide confidence intervals reported in Tables 2 and 3.

### Supplementary Information

Supplementary Information.

## Data Availability

The data for this study were provided by Thailand’s Bureau of Registration Administration (BORA). Access requests for the data (along with the code for this study) can be channeled to BORA through plapitan@adb.org.
