# Supplementary material for: Mixed effects modelling of excess mortality and COVID-19 lockdowns in Thailand
Source: Sci Rep. 2024 Apr 8;14:8240. doi: 10.1038/s41598-024-58358-3 (PMC11001903; doi:10.1038/s41598-024-58358-3)
Supplement: Supplementary file 1 — Supplementary Information. [file 41598_2024_58358_MOESM1_ESM.docx]

# Appendix: Additional data details and tables

**Facebook mobility measure (*StayPut)***

Movement range data are downloadable from The Humanitarian Data Exchange. Two variables based on Facebook user data are available:

1. Change in movement: the count of how many tiles that an average user in a population appears in over time. This measure is not used in the study.
2. Stay Put metric (*StayPut*): the proportion of a population that did not move outside of a 0.6 square kilometer area over a 24-hour period. This is averaged at the province-month level for Thailand and used in the study.

**Table A1. Relation between Facebook mobility indicator and lockdown stringency**

|  | **(1)**  ***Stay Put*** |
| --- | --- |
| Red zone | 0.0121*** |
|  | (0.0023) |
| Yellow zone | -0.0003 |
|  | (0.0012) |
| Observations | 1,001 |
| R-squared | 0.9342 |
| Within R-squared | 0.1382 |
| Province FE | 77 |
| Month-Year FE | 12 |

Note: This table uses data on color-coded zones used by the government between December 2020-Decmeber 2021. The excluded code is `Green Zone’. $Stay Put$ is a proxy variable capturing mobility restrictions, a monthly aggregate at the province level of the fraction of Facebook users who stayed within one 0.6-square km area throughout the day. We include month-year and province fixed effects, to account for any province or month level characteristics that are specific to the province or month such as population and weather. Standard errors are in parentheses. We cluster errors at the province level. Level of significance: *** 0.01 ** 0.05 * 0.1

**Table A2. Mobility and excess mortality**

|  | **Excess Mortality** | | **Adjusted P-score** | |  |
| --- | --- | --- | --- | --- | --- |
|  |  | |  | |  |
| Stay Put | 2895.3470** | | 209.3605*** | |  |
|  | (1312.5509) | | (58.9807) | |  |
| Lag Stay Put (1) | | -584.5387 | | 28.9119 | |
|  | (829.5815) | | (54.6689) | |  |
| Lag Stay Put (2) | -360.0722 | | -21.5347 | |  |
|  | (744.3209) | | (54.5118) | |  |
| Lag outcome | 0.6524*** | | 0.3391*** | |  |
|  | (0.1919) | | (0.0668) | |  |
| Observations | 1,540 | | 1,540 | |  |
| R-squared | 0.5379 | | 0.3827 | |  |
| Province FE | Yes | | Yes | |  |
| Month-year FE | Yes | | Yes | |  |

Note: This table presents results from a linear ARDL model with one lag of dependent and two lags of the explanatory variable. Standard errors are in parentheses. We cluster standard errors at the province level and include month-year and province fixed effects. Level of significance: ***0.01, **0.05, *0.1.

**Table A3. Mobility and excess mortality during four waves**

|  | **Excess Mortality** | **Adjusted P-score** |
| --- | --- | --- |
|  |  |  |
| Stay Put | 2331.1334** | 180.8173*** |
|  | (971.6861) | (48.1135) |
| Wave=1 x Lag Stay Put | -647.0145 | 10.6500 |
|  | (601.3372) | (46.0879) |
| Wave=2 x Lag Stay Put | -364.7109 | 64.0256 |
|  | (543.3500) | (55.0596) |
| Wave=3 x Lag Stay Put | -583.2166 | -30.4936 |
|  | (733.2607) | (54.0417) |
| Wave=4 x Lag Stay Put | -308.1650 | 54.4882 |
|  | (355.9503) | (48.7310) |
| Lag outcome | 0.6484*** | 0.3337*** |
|  | (0.1916) | (0.0649) |
| Observations | 1,617 | 1,617 |
| R-squared | 0.5291 | 0.3912 |
| Province FE | Yes | Yes |
| Month-year FE | Yes | Yes |

Standard errors are in parentheses.

**Figure A1. Predicted and actual deaths, Jan 2020-December 2021**

**Figure A2. Population Pyramid of Thailand, 2019**


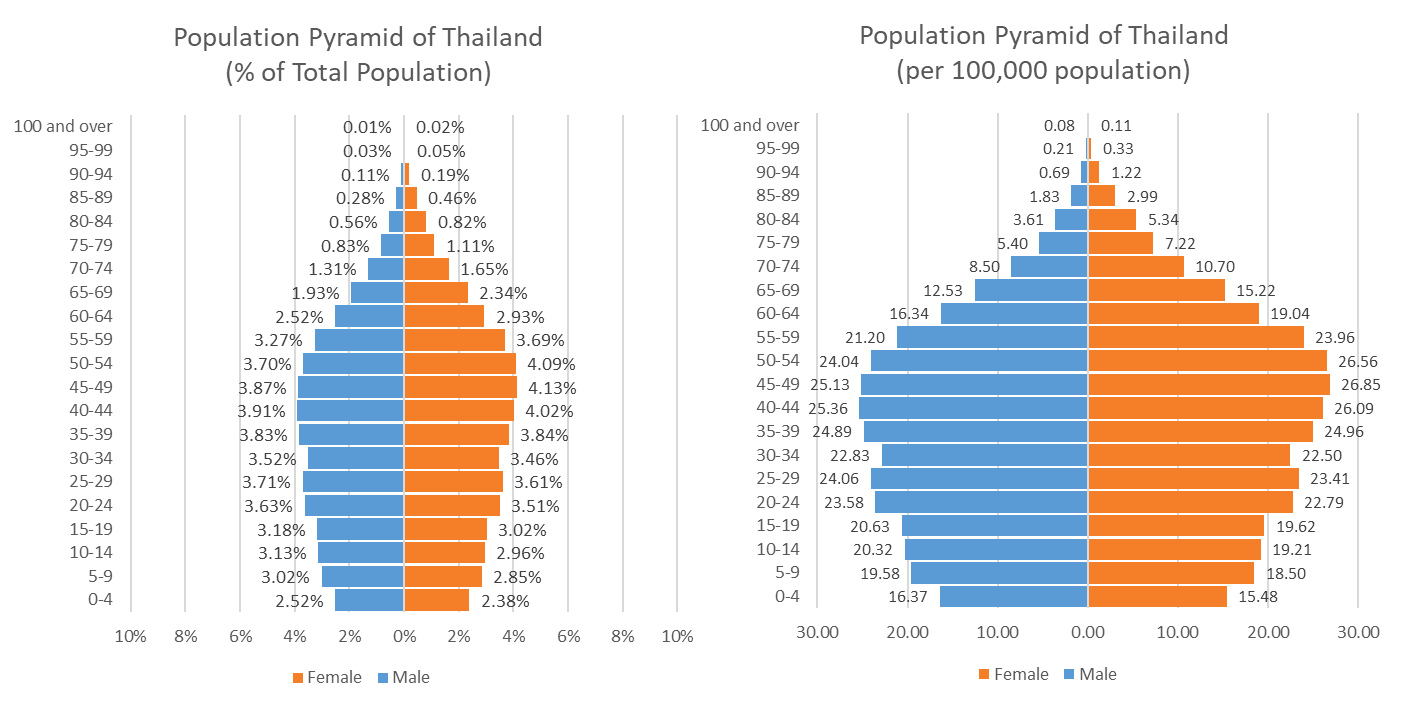


Source: Authors’ estimates based on NSO of Thailand population data accessed 13 November 2020.

**Table A4a. Regression predicting 2020 deaths using Fixed Effects Negative Binomial model with population as covariate**

|  | (1) | (2) | (3) | (4) |
| --- | --- | --- | --- | --- |
| VARIABLES | Female –  0 to 14 years | Male –  0 to 14 years | Female –  65+ years | Male –  65+ years |
| Month trend | 0.000584*** | 0.000881*** | 0.00183*** | 0.00231*** |
|  | (0.000120) | (8.77e-05) | (0.000129) | (0.000130) |
| Feb | -0.139*** | -0.156*** | -0.145*** | -0.130*** |
|  | (0.0103) | (0.00752) | (0.00887) | (0.00848) |
| March | -0.0834*** | -0.104*** | -0.141*** | -0.127*** |
|  | (0.0127) | (0.00923) | (0.0109) | (0.0104) |
| April | -0.0732*** | -0.0807*** | -0.116*** | -0.103*** |
|  | (0.0142) | (0.0103) | (0.0121) | (0.0116) |
| May | -0.0703*** | -0.106*** | -0.165*** | -0.146*** |
|  | (0.0132) | (0.00966) | (0.0114) | (0.0109) |
| June | -0.101*** | -0.157*** | -0.240*** | -0.212*** |
|  | (0.0120) | (0.00881) | (0.0104) | (0.00999) |
| July | -0.0730*** | -0.126*** | -0.185*** | -0.168*** |
|  | (0.0112) | (0.00823) | (0.00973) | (0.00931) |
| August | -0.0215** | -0.101*** | -0.135*** | -0.131*** |
|  | (0.0108) | (0.00798) | (0.00940) | (0.00902) |
| September | -0.0780*** | -0.147*** | -0.160*** | -0.154*** |
|  | (0.0108) | (0.00794) | (0.00935) | (0.00898) |
| October | -0.0216** | -0.0832*** | -0.0848*** | -0.0881*** |
|  | (0.0104) | (0.00768) | (0.00903) | (0.00871) |
| November | -0.0736*** | -0.109*** | -0.130*** | -0.126*** |
|  | (0.0103) | (0.00748) | (0.00888) | (0.00853) |
| December | -0.0582*** | -0.0793*** | -0.0653*** | -0.0645*** |
|  | (0.00988) | (0.00721) | (0.00851) | (0.00816) |
| Temperature | 0.00898*** | 0.00966*** | 0.0185*** | 0.0136*** |
|  | (0.00213) | (0.00155) | (0.00183) | (0.00175) |
| Population | 7.63e-07*** | 8.34e-07*** | 1.04e-06*** | 1.20e-06*** |
|  | (2.16e-07) | (1.88e-07) | (2.93e-07) | (4.40e-07) |
| Constant | 5.437*** | 5.481*** | 4.576*** | 4.920*** |
|  | (0.187) | (0.120) | (0.0666) | (0.0716) |
|  |  |  |  |  |
| Observations | 4,620 | 4,620 | 4,620 | 4,620 |
| Province FEs | Y | Y | Y | Y |
| Month-year FEs | Y | Y | Y | Y |

Standard errors in parentheses

*** p<0.01, ** p<0.05, * p<0.1

**Table A4b. Regression predicting 2021 deaths using Fixed Effects Negative Binomial model with population as covariate**

|  | (1) | (2) | (3) | (4) |
| --- | --- | --- | --- | --- |
| VARIABLES | Female –  0 to 14 years | Male –  0 to 14 years | Female –  65+ years | Male –  65+ years |
| Month trend | 0.00153*** | 0.00166*** | 0.00318*** | 0.00378*** |
|  | (0.000114) | (8.26e-05) | (0.000115) | (0.000117) |
| Feb | -0.115*** | -0.142*** | -0.138*** | -0.140*** |
|  | (0.0128) | (0.00920) | (0.0103) | (0.0101) |
| March | -0.0277* | -0.0245** | -0.0710*** | -0.0784*** |
|  | (0.0155) | (0.0111) | (0.0125) | (0.0123) |
| April | -0.0703*** | -0.0742*** | -0.117*** | -0.121*** |
|  | (0.0164) | (0.0118) | (0.0131) | (0.0129) |
| May | -0.0395** | -0.0623*** | -0.125*** | -0.134*** |
|  | (0.0161) | (0.0116) | (0.0131) | (0.0128) |
| June | -0.0594*** | -0.106*** | -0.201*** | -0.193*** |
|  | (0.0144) | (0.0104) | (0.0118) | (0.0116) |
| July | -0.0264* | -0.0828*** | -0.166*** | -0.150*** |
|  | (0.0137) | (0.00992) | (0.0113) | (0.0110) |
| August | 0.0503*** | -0.0239** | -0.0789*** | -0.0793*** |
|  | (0.0133) | (0.00967) | (0.0109) | (0.0107) |
| September | -0.0445*** | -0.103*** | -0.143*** | -0.154*** |
|  | (0.0133) | (0.00961) | (0.0108) | (0.0107) |
| October | -0.00424 | -0.0588*** | -0.0791*** | -0.0938*** |
|  | (0.0126) | (0.00910) | (0.0102) | (0.0101) |
| November | -0.0312** | -0.0674*** | -0.0850*** | -0.0917*** |
|  | (0.0125) | (0.00897) | (0.0101) | (0.00989) |
| December | -0.0279** | -0.0434*** | -0.0475*** | -0.0506*** |
|  | (0.0124) | (0.00885) | (0.00992) | (0.00972) |
| Temperature | 0.000631 | -0.00111 | 0.00507** | 0.00394* |
|  | (0.00256) | (0.00185) | (0.00211) | (0.00206) |
| Population | -2.77e-07*** | -2.72e-07*** | -3.68e-07 | -1.98e-06*** |
|  | (9.00e-08) | (1.01e-07) | (3.24e-07) | (5.04e-07) |
| Constant | 4.600*** | 5.137*** | 4.329*** | 4.496*** |
|  | (0.0938) | (0.0787) | (0.0651) | (0.0668) |
|  |  |  |  |  |
| Observations | 4,620 | 4,620 | 4,620 | 4,620 |
| Province FEs | Y | Y | Y | Y |
| Month-year FEs | Y | Y | Y | Y |

Standard errors in parentheses

*** p<0.01, ** p<0.05, * p<0.1

**Table A5a.** **Regression predicting 2020 deaths using Mixed Effects model
with province random effects and population as a covariate**

|  | (1) | (2) | (3) | (4) |
| --- | --- | --- | --- | --- |
| VARIABLES | Female –  0 to 14 years | Male –  0 to 14 years | Female –  65+ years | Male –  65+ years |
| Month trend | 0.000640*** | 0.000899*** | 0.00185*** | 0.00236*** |
|  | (0.000108) | (7.32e-05) | (9.15e-05) | (9.80e-05) |
| Feb | -0.139*** | -0.156*** | -0.146*** | -0.128*** |
|  | (0.00939) | (0.00628) | (0.00600) | (0.00612) |
| March | -0.0831*** | -0.102*** | -0.147*** | -0.125*** |
|  | (0.0115) | (0.00769) | (0.00741) | (0.00757) |
| April | -0.0723*** | -0.0789*** | -0.116*** | -0.103*** |
|  | (0.0129) | (0.00865) | (0.00834) | (0.00852) |
| May | -0.0694*** | -0.104*** | -0.168*** | -0.146*** |
|  | (0.0120) | (0.00806) | (0.00777) | (0.00794) |
| June | -0.101*** | -0.155*** | -0.244*** | -0.212*** |
|  | (0.0109) | (0.00735) | (0.00713) | (0.00726) |
| July | -0.0737*** | -0.125*** | -0.189*** | -0.169*** |
|  | (0.0102) | (0.00686) | (0.00663) | (0.00677) |
| August | -0.0222** | -0.100*** | -0.141*** | -0.133*** |
|  | (0.00983) | (0.00666) | (0.00640) | (0.00655) |
| September | -0.0783*** | -0.148*** | -0.162*** | -0.153*** |
|  | (0.00979) | (0.00663) | (0.00635) | (0.00650) |
| October | -0.0219** | -0.0825*** | -0.0867*** | -0.0849*** |
|  | (0.00948) | (0.00640) | (0.00613) | (0.00628) |
| November | -0.0738*** | -0.109*** | -0.131*** | -0.123*** |
|  | (0.00930) | (0.00624) | (0.00602) | (0.00615) |
| December | -0.0594*** | -0.0789*** | -0.0629*** | -0.0619*** |
|  | (0.00897) | (0.00599) | (0.00575) | (0.00588) |
| Temperature | 0.00887*** | 0.00921*** | 0.0201*** | 0.0142*** |
|  | (0.00193) | (0.00129) | (0.00124) | (0.00126) |
| Population | 1.71e-06*** | 1.71e-06*** | 6.58e-07*** | 7.69e-07*** |
|  | (1.50e-07) | (1.58e-07) | (1.63e-07) | (2.64e-07) |
| Constant | 3.211*** | 4.024*** | 4.309*** | 4.408*** |
|  | (0.0792) | (0.0729) | (0.0785) | (0.0771) |
| Variance of province intercept | -0.939*** | -0.904*** | -0.457*** | -0.484*** |
|  | (0.0854) | (0.0884) | (0.0812) | (0.0815) |
| Observations | 4,620 | 4,620 | 4,620 | 4,620 |
| Province FEs | Y | Y | Y | Y |
| Month-year FEs | Y | Y | Y | Y |

Standard errors in parentheses

*** p<0.01, ** p<0.05, * p<0.1

**Table A5b. Regression predicting 2021 deaths using Mixed Effects model
with province random effects and population as a covariate**

|  | (1) | (2) | (3) | (4) |
| --- | --- | --- | --- | --- |
| VARIABLES | Female –  0 to 14 years | Male –  0 to 14 years | Female –  65+ years | Male –  65+ years |
| Month trend | 0.00176*** | 0.00176*** | 0.00275*** | 0.00309*** |
|  | (5.60e-05) | (5.60e-05) | (6.80e-05) | (7.25e-05) |
| Feb | -0.142*** | -0.142*** | -0.141*** | -0.134*** |
|  | (0.00613) | (0.00613) | (0.00577) | (0.00587) |
| March | -0.0249*** | -0.0249*** | -0.0810*** | -0.0760*** |
|  | (0.00745) | (0.00745) | (0.00708) | (0.00723) |
| April | -0.0736*** | -0.0736*** | -0.117*** | -0.117*** |
|  | (0.00794) | (0.00794) | (0.00751) | (0.00766) |
| May | -0.0609*** | -0.0609*** | -0.131*** | -0.127*** |
|  | (0.00781) | (0.00781) | (0.00742) | (0.00757) |
| June | -0.104*** | -0.104*** | -0.202*** | -0.184*** |
|  | (0.00702) | (0.00702) | (0.00671) | (0.00683) |
| July | -0.0669*** | -0.0669*** | -0.152*** | -0.128*** |
|  | (0.00663) | (0.00663) | (0.00633) | (0.00644) |
| August | -0.0128** | -0.0128** | -0.0613*** | -0.0510*** |
|  | (0.00648) | (0.00648) | (0.00615) | (0.00626) |
| September | -0.103*** | -0.103*** | -0.139*** | -0.136*** |
|  | (0.00644) | (0.00644) | (0.00609) | (0.00621) |
| October | -0.0607*** | -0.0607*** | -0.0751*** | -0.0801*** |
|  | (0.00608) | (0.00608) | (0.00573) | (0.00586) |
| November | -0.0697*** | -0.0697*** | -0.0827*** | -0.0769*** |
|  | (0.00599) | (0.00599) | (0.00566) | (0.00576) |
| December | -0.0468*** | -0.0468*** | -0.0408*** | -0.0403*** |
|  | (0.00591) | (0.00591) | (0.00558) | (0.00568) |
| Temperature | -0.00177 | -0.00177 | 0.00810*** | 0.00537*** |
|  | (0.00125) | (0.00125) | (0.00118) | (0.00120) |
| Population | 4.16e-07*** | 4.16e-07*** | 1.52e-06*** | 2.32e-06*** |
|  | (1.38e-07) | (1.38e-07) | (1.18e-07) | (1.94e-07) |
| Constant | 4.650*** | 4.650*** | 4.555*** | 4.573*** |
|  | (0.0861) | (0.0861) | (0.0744) | (0.0713) |
| Variance of province intercept | -0.501*** | -0.501*** | 0.00275*** | -0.567*** |
|  | (0.0919) | (0.0919) | (6.80e-05) | (0.0813) |
| Observations | 4,620 | 4,620 | 4,620 | 4,620 |
| Province REs | Y | Y | Y | Y |
| Month FEs | Y | Y | Y | Y |

Standard errors in parentheses

*** p<0.01, ** p<0.05, * p<0.1
